# Supplementary material for: Polyketide synthase gene clusters in the ericoid mycorrhizal fungus Oidiodendron maius: genome-wide prediction, expression profiling, and functional characterization of knockout mutants
Source: Front Plant Sci. 2026 Sep 16;17:1924430. doi: 10.3389/fpls.2026.1924430 (PMC13623903; doi:10.3389/fpls.2026.1924430)
Supplement: Supplementary file 1 [file SupplementaryFile1.zip › Supplementary Materials/Supplementary Folder 1.zip!/Oidma1_AssemblyScaffolds_Repeatmasked/knownclusterblast/cluster51/jgi.p_Oidma1_35762_mibig_hits.html]

| MIBiG Protein | Description | MIBiG Cluster | MiBiG Product | % ID | % Coverage | BLAST Score | E-value |
| --- | --- | --- | --- | --- | --- | --- | --- |
| ADI24928.1 | VrtC | BGC0000168 | Viridicatumtoxin\_biosynthetic\_gene\_cluster | 49 | 91.4893617021 | 414 | 2e-115 |
| EGD99349.1 | dimethylallyl\_tryptophan\_synthase | BGC0001144 | Neosartoricin\_biosynthetic\_gene\_cluster | 42 | 90.0 | 361 | 2e-99 |
| AGA37265.1 | prenyltransferase | BGC0000816 | Malbrancheamide\_biosynthetic\_gene\_cluster | 23 | 90.6382978723 | 96 | 7e-20 |
